# Unexplained Leg Swelling Leading to a Diagnosis of Hepatocellular Carcinoma: A Case Report

**DOI:** 10.7759/cureus.84206

**Published:** 2025-05-16

**Authors:** Nur Athirah Abd Rasid, Noor Azimah Muhammad

**Affiliations:** 1 Department of Family Medicine, Universiti Kebangsaan Malaysia Medical Centre, Kuala Lumpur, MYS

**Keywords:** atypical presentation, hepatocellular carcinoma, leg swelling, limb edema, peripheral edema

## Abstract

Hepatocellular carcinoma (HCC) typically presents with abdominal pain, jaundice, or hepatic decompensation. However, peripheral edema as an initial presentation is uncommon and may lead to diagnostic delays. Here, we report the case of a 77-year-old woman with underlying diabetes mellitus, hypertension, and dyslipidemia who presented with bilateral leg swelling. Cardiac and renal evaluations were unremarkable. Liver function tests revealed mild derangement. Further investigations, including abdominal imaging and elevated alpha-fetoprotein levels, confirmed the diagnosis of HCC. This case highlights the importance of considering hepatic malignancy in patients presenting with unexplained peripheral edema. A comprehensive clinical evaluation and timely imaging are crucial for early diagnosis and improved patient outcomes.

## Introduction

Bilateral lower limb edema is a common clinical presentation in elderly patients. Evaluating bilateral lower limb edema in the elderly poses unique challenges as they have multiple comorbidities, leading to overlapping clinical presentations that complicate the diagnosis. Systemic diseases, such as heart failure and renal failure, are the most common causes of bilateral lower limb edema in this population [1]. Hypoproteinemia from liver failure is another important cause to consider [2]. Another common cause is chronic venous insufficiency, a localized problem related to valve incompetence, causing fluid accumulation in the affected limb [1]. Medications such as calcium channel blockers, non-steroidal anti-inflammatory drugs, and steroids can also be the cause in elderly patients with multiple comorbidities, including diabetes, and on polypharmacy [2,3].

On the other hand, peripheral edema is an uncommon initial presentation of hepatocellular carcinoma (HCC) [4]. HCC is the most common type of primary liver cancer, accounting for approximately 85-90% of cases [5,6]. The risk factors for HCC include chronic hepatitis B virus or hepatitis C virus infections, excessive alcohol consumption, non-alcoholic fatty liver disease, and exposure to environmental toxins such as aflatoxins [6,7]. Clinically, HCC presents with a wide spectrum of symptoms, ranging from abdominal pain and hepatic dysfunction [8,9]. Advancements in imaging techniques, such as CT and MRI, have facilitated earlier detection of HCC, even in asymptomatic patients [9]. Alpha-fetoprotein (AFP) is used as a tumor marker in the evaluation of HCC, but it should not be used in isolation for diagnosis due to its limited sensitivity and specificity [10].

This case report highlights the diagnostic challenges in diagnosing HCC in the elderly presenting with bilateral lower limb edema. By detailing this patient’s case, this case report aims to highlight the importance of a thorough evaluation of lower limb edema as a non-specific presentation of HCC.

## Case presentation

A 77-year-old woman presented for the first time to our primary care setting with bilateral leg swelling and reduced effort tolerance for four months. The leg swelling improved with leg elevation and diuretics that were prescribed at multiple emergency department visits. She had type 2 diabetes mellitus (diagnosed 32 years ago), hypertension (for 30 years), and dyslipidemia (for 30 years), managed in another healthcare clinic. She was on five oral medications, including metformin, gliclazide, hydrochlorothiazide, perindopril, and simvastatin. She reported good adherence to the treatment. Most recent results showed controlled type 2 diabetes (HbA1c: 6.6%) and blood pressure (132/71 mmHg). However, her dyslipidemia remained suboptimally controlled, with a low-density lipoprotein level of 3.43 mmol/L and a high-density lipoprotein 0.71 mmol/L. She denied the use of supplemental medicines or remedies, had no prior surgeries or blood transfusions, and reported no known exposure to environmental toxins, including aflatoxins.

She did not smoke cigarettes or drink alcohol, and led a healthy and active lifestyle. Clinical examination revealed a well-hydrated elderly patient with a body mass index of 27.2 kg/m^2^, stable vital signs, and bilateral pitting lower limb edema up to mid-shins. The respiratory and cardiovascular examinations were unremarkable. There were no peripheral signs of chronic liver disease or hepatosplenomegaly. Urinalysis was normal with no proteinuria.

Laboratory investigation showed slight hypoalbuminemia and mildly raised aspartate transaminase and alkaline phosphatase (Table 1). No baseline laboratory investigations were documented before the presentation. Other parameters, including renal profile, urine protein creatinine index, and N-terminal pro-B-type natriuretic peptide, were normal. An echocardiogram showed a normal heart with an ejection fraction of 63%. Hence, renal or cardiac causes were unlikely. Screening for hepatitis B and C viruses was non-reactive.

**Table 1:** Summary of laboratory investigations. NT-proBNP: N-terminal pro-B-type natriuretic peptide; HDL: high-density lipoprotein; LDL: low-density lipoprotein; NHDL: non-high-density lipoprotein cholesterol; UPCI: urine protein creatinine ratio; HCV: hepatitis C virus; AFP: alpha-fetoprotein

Parameter	Results	Normal range
Renal profile		
Urea (mmol/L)	2.8	2.5–6.7
Sodium (mmol/L)	137	135–145
Potassium (mmol/L)	4.1	3.5–5.1
Creatinine (µmol/L)	37.5	49–90
Liver function test		
Total protein (g/L)	60	64–83
Albumin (g/L)	30	34–48
Alkaline phosphatase (U/L)	208	40–150
Aspartate transaminase (U/L)	64	5–34
Alanine transaminase (U/L)	17	0–55
Total bilirubin (µmol/L)	10.9	3.4–20.5
NT-proBNP (pg/mL)	88	>125
Fasting lipid profile		
Total cholesterol (mmol/L)	4.81	<5.2
HDL (mmol/L)	0.71	1.55–3
LDL (mmol/L)	3.43	<3.80
NHDL (mmol/L)	4.10	Not applicable
Triglycerides (mmol/L)	1.47	<1.7
HbA1c (%)	6.6	<5.7%
UPCI (g/mmol creatinine)	0.02	<0.02
HBs antigen	Non-reactive	-
AntiHCV	Non-reactive	-
AFP (ng/mL)	62.91	0.00–8.78

**Figure 1:**
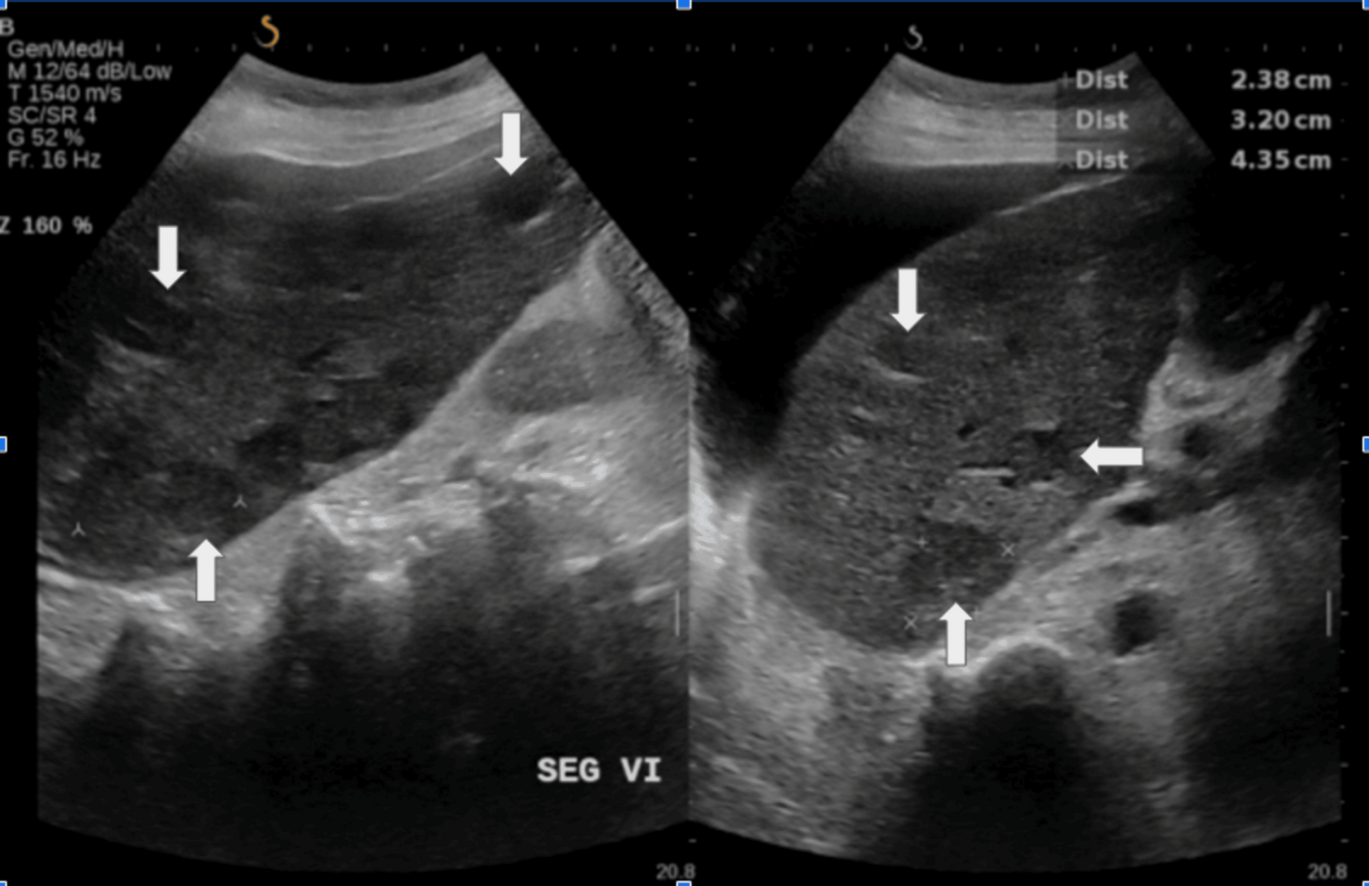
Ultrasound of the hepatobiliary system showing multiple hypoechoic liver lesions (largest 4.0 × 3.2 cm) in the background of liver cirrhosis.

**Figure 2:**
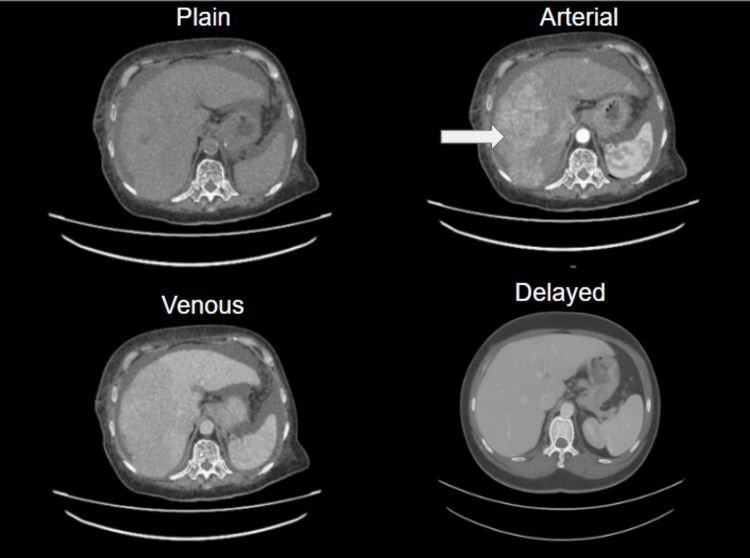
Four-phase contrast-enhanced CT images of the liver demonstrating multiple arterially enhancing lesions of varying sizes scattered in both liver lobes predominantly in the right lobe, showing washout on the portovenous and delayed phase.

Ultrasound of the hepatobiliary system (Figure 1) showed liver cirrhosis with multiple lesions of varying sizes suggestive of HCC, and was further confirmed by a four-phase CT of the liver (Figure 2) and significantly raised AFP. Meanwhile, CT-TAP revealed no evidence of metastasis. The Child-Pugh Score was calculated to be 8 (Class B) based on an international normalized ratio of 1.5, albumin of 30 g/L, bilirubin of 10.9 µmol/L, and moderate ascites seen on imaging. The final diagnosis was sub-classified according to the Modified Barcelona Clinic Liver Cancer (BCLC) staging system. The patient had multifocal HCC in a cirrhotic liver, with a performance status of Eastern Cooperative Oncology Group 2 and moderate ascites, consistent with intermediate to advanced-stage disease.

She was initiated on lenvatinib 8 mg once daily. However, after only five doses, the medication was discontinued due to generalized fatigue, anorexia, and malaise, suggestive of early drug intolerance. Given these symptoms and her underlying Child-Pugh Class B liver function, she was deemed unsuitable for further systemic therapy and was transitioned to best supportive care.

The patient was followed up regularly for symptom management and palliative support. Despite conservative measures, her condition progressively deteriorated. She eventually passed away five months after the definitive diagnosis.

## Discussion

This is a case of an elderly woman with multiple chronic medical conditions who presented with non-specific symptoms of leg swelling and fatigue. These symptoms, in the context of polypharmacy and her comorbidities, delayed the suspicion of an underlying malignancy and posed a diagnostic challenge. Renal or cardiac pathology and medication side effects were important etiologies of the lower limb edema to be considered. Peripheral edema is an atypical manifestation of HCC, and a few cases have been reported in the literature [4,11].

Lower limb edema may be related to liver dysfunction and hypoalbuminemia. A decline in serum albumin causes reduced oncotic pressure and facilitates fluid extravasation into the interstitial space, resulting in peripheral edema. This patient had mild hypoalbuminemia of 30 g/L at presentation, serving as a plausible contributing mechanism of symptoms. However, the absence of other clinical findings, such as ascites, abdominal mass, or peripheral signs of chronic liver disease, delayed the clinical suspicion of a hepatic cause. A wide spectrum of clinical presentations has made establishing an HCC diagnosis challenging [8].

Another plausible mechanism for lower limb edema in HCC includes inferior vena cava obstruction and venous congestion because of tumour invasion [4,11]. However, the imaging studies of this patient showed no significant compression or obstruction in the inferior vena cava. Often, HCC is an incidental finding in 20-30% of patients with liver cirrhosis or chronic liver disease [9]. However, the patient had no apparent risk factors for HCC, including hepatitis virus infection, and previous laboratory investigations were not available for review, limiting the ability for early recognition of HCC.

Despite the absence of traditional risk factors, this patient had long-standing diabetes and dyslipidemia, which are known to be associated with metabolic dysfunction-associated steatotic liver disease that can progress to non-alcoholic steatohepatitis, cirrhosis, and, subsequently, HCC [6,7]. Otherwise, she denied consuming any supplemental medicines or remedies, which raises the possibility of exposure to hepatotoxic agents, which are linked to HCC development [12]. Routine surveillance in high-risk patients is critical for the early detection of HCC. Guidelines recommend six-monthly ultrasonography with or without AFP measurement [13]. This patient had no indication for such a surveillance procedure, leading to a missed opportunity for earlier intervention. Her final diagnosis was sub-classified according to BCLC staging as advanced HCC with Child-Pugh Class B, indicating a moderately severe level of liver dysfunction [14].

Although the diagnosis of HCC was made at an advanced stage, this case demonstrates a structured diagnostic workup in patients presenting with atypical symptoms. Initial laboratory investigations with mildly deranged liver function had prompted further investigations. Ultrasound of the hepatobiliary system and four-phase CT of the liver pointed toward multifocal HCC. Histopathological examination of the liver tissue was not performed, as biopsy is often reserved for cases with diagnostic uncertainty and to minimize the risk of tumor seeding [15].

The patient’s management aligns with current evidence-based guidelines. She was initiated on lenvatinib, a tyrosine kinase inhibitor that has demonstrated non-inferiority to sorafenib in improving overall survival in advanced HCC [16]. The prognosis of HCC is influenced by tumour stage, liver function, and treatment accessibility. Advanced HCC, as seen in this patient, carries a guarded prognosis with a median survival of 6-12 months without treatment [17].

## Conclusions

This case underscores the need for primary care and emergency clinicians to maintain a high index of suspicion for HCC as a differential diagnosis for bilateral lower limb edema, especially in elderly patients with multiple chronic diseases. Symptomatic treatment with diuretics should just be a temporary measure. While HCC surveillance in those with chronic liver disease or dysfunction is recommended, such surveillance in patients with chronic medical illnesses calls for more evidence.

## References

[1] Han S, Kee Y, Moon S (2014). Etiologies and underlying diseases of leg edema in elderly patients. J Korean Geriatr Soc.

[2] Largeau B, Cracowski JL, Lengellé C, Sautenet B, Jonville-Béra AP (2021). Drug-induced peripheral oedema: an aetiology-based review. Br J Clin Pharmacol.

[3] Thaler HW, Wirnsberger G, Pienaar S, Roller RE (2010). Bilateral leg edema in the elderly. Clinical considerations and treatment options. Eur Geriatr Med.

[4] Mengi S, Phelps L, Silva TS, Ludman A (2019). Clinical examination remains crucial to the correct diagnosis: a case of severe peripheral oedema referred for investigation of heart failure. BMJ Case Rep.

[5] Lafaro KJ, Demirjian AN, Pawlik TM (2015). Epidemiology of hepatocellular carcinoma. Surg Oncol Clin N Am.

[6] Rawla P, Sunkara T, Muralidharan P, Raj JP (2018). Update in global trends and aetiology of hepatocellular carcinoma. Contemp Oncol (Pozn).

[7] Baffy G, Brunt EM, Caldwell SH (2012). Hepatocellular carcinoma in non-alcoholic fatty liver disease: an emerging menace. J Hepatol.

[8] Dimitroulis D, Damaskos C, Valsami S (2017). From diagnosis to treatment of hepatocellular carcinoma: an epidemic problem for both developed and developing world. World J Gastroenterol.

[9] Di Bisceglie AM (2002). Epidemiology and clinical presentation of hepatocellular carcinoma. J Vasc Interv Radiol.

[10] Hanif H, Ali MJ, Susheela AT, Khan IW, Luna-Cuadros MA, Khan MM, Lau DT (2022). Update on the applications and limitations of alpha-fetoprotein for hepatocellular carcinoma. World J Gastroenterol.

[11] Chen M, Huang X, Yang Q (2019). Hepatocellular carcinoma with inferior vena cava and right atrial tumor thrombus: a case report. Echocardiography.

[12] Gomaa AI, Khan SA, Toledano MB, Waked I, Taylor-Robinson SD (2008). Hepatocellular carcinoma: epidemiology, risk factors and pathogenesis. World J Gastroenterol.

[13] Chidambaranathan-Reghupaty S, Fisher PB, Sarkar D (2021). Hepatocellular carcinoma (HCC): epidemiology, etiology and molecular classification. Adv Cancer Res.

[14] Johnson PJ, Pinato DJ, Kalyuzhnyy A, Toyoda H (2022). Breaking the Child-Pugh dogma in hepatocellular carcinoma. J Clin Oncol.

[15] Charach L, Zusmanovitch L, Charach G (2017). Hepatocellular carcinoma. Part 2: clinical presentation and diagnosis. EMJ Hepatol.

[16] Qian Y, Gong L, Li S, Mao K, Li X, Liao G (2022). Case report: radiotherapy plus immunotherapy and lenvatinib for the treatment of recurrent hepatocellular carcinoma with a right atrium and inferior vena cava tumor thrombus. Front Oncol.

[17] Greten TF, Lai CW, Li G, Staveley-O'Carroll KF (2019). Targeted and immune-based therapies for hepatocellular carcinoma. Gastroenterology.

